# Comparative study of subtalar arthrodesis after calcaneal frature malunion with autologous bone graft or freeze-dried xenograft

**DOI:** 10.1186/s40634-015-0024-2

**Published:** 2015-05-07

**Authors:** Carlo Henning, Gabriel Poglia, Murilo Anderson Leie, Carlos Roberto Galia

**Affiliations:** Department of Orthopaedics, Hospital de Clínicas de Porto Alegre, Medicine School, Universidade Federal do Rio Grande do Sul, Rua Ramiro Barcelos, 2350, Zip Code 90035-003 Porto Alegre Rio Grande do Sul, Brazil

**Keywords:** Calcaneus, Malunion, Arthrodesis, Bone graft, Xenograft, Freeze-dried

## Abstract

**Background:**

Calcaneal fracture malunion may evolve into arthrosis and severe foot deformities. The aim of this study was to identify differences in bony union following corrective subtalar arthrodesis with interposition of autologous tricortical bone graft or freeze-dried bovine xenograft.

**Methods:**

We prospectively evaluated 12 patients who underwent subtalar arthrodesis, six patients received autografts and 6 received freeze-dried bovine xenografts. After a mean followup of 58 weeks, the patients were clinical assessed using AOFAS scale and the visual analog scale (VAS) for pain and for final radiographic parameters measurement. Two blind raters evaluated the length of time required for solid union of the arthrodesis and graft integration by retrospective radiographic examination.

**Results:**

In the autograft group, AOFAS score improved from a preoperative average of 37 to 64 points postoperatively (p = 0.02) and mean VAS score improved from 4.7 to 1.9 (p = 0.028). In the xenograft group, AOFAS score improved from 38 to 74 points (p = 0.02) and VAS from 5.5 to 2.7 (p = 0.046). Solid union was achieved in all cases in the autograft group at an average of 5.3 weeks and in five cases in the xenograft group at 8.8 weeks (p = 0.077). Graft integration occurred after an average of 10.7 weeks in the autograft group and 28.8 weeks in the xenograft group (p = 0.016).

**Conclusion:**

With the numbers available, no significant difference could be detected in the length of time required for solid union of subtalar arthrodesis between groups, although time to integration of freeze-dried bovine xenografts was statistically higher. Clinical and functional improvement was observed in both groups.

## Background

Late complications of calcaneal fracture malunion are related to: arthrosis of the talocalcaneal joint, and possibly of the calcaneocuboid joint, due to joint incongruity or chondral lesion; widening of the calcaneus, which may lead to changes in the peroneal tendons, calcaneofibular impingement, and shoe wear difficulties; and often varus deformity of the calcaneus, loss of hindfoot height, and foot flattening (Chandler et al. 1999; Nickisch and Anderson 2006). In addition, neurovascular and heel pad injuries may be responsible for chronic pain and functional limitation in these patients (Lim and Leung 2001).

Several authors have described subtalar arthrodesis with bone graft block interposition in the treatment of posttraumatic arthrosis of this joint along with lateral calcaneal wall prominence resection in order to correct deformities and hindfoot malalignment with good clinical and radiographic outcomes (Amendola and Lammens 1996; Bednarz et al. 1997; Burton et al. 1998; Chan and Alexander 1997; Chen et al. 1998; Clare et al. 2005; Easley et al. 2000; Garras et al. 2008; Marti et al. 1999; Myerson and Quill 1993; Rammelt et al. 2004; Thermann et al. 1999; Trnka et al. 2001).

Solid union of this type of subtalar arthrodesis using autologous tricortical iliac crest graft has been achieved in 86 to 100% of cases (Amendola and Lammens 1996; Bednarz et al. 1997; Burton et al. 1998; Chan and Alexander 1997; Chen et al. 1998; Marti et al. 1999; Myerson and Quill 1993; Rammelt et al. 2004; Thermann et al. 1999). However, complications associated with bone graft harvest from the iliac crest have been estimated to occur in up to 49% of cases (Finkemeier 2002; Seiler and Johnson 2000). The most common donor site complications include chronic pain, nerve injury, hematoma and infection (Scranton 2002). Furthermore, it is an additional procedure that may increase operative time, length of hospital stay and costs (Salama 1983; Seiler and Johnson 2000). With the use of allografts in this type of arthrodesis, union rates may vary considerably, from 20 to 90.5% (Easley et al. 2000; Garras et al. 2008; Seiler and Johnson 2000). No studies using xenografts in this particular type of surgery were identified by the authors in the literature.

The need to use large quantities of bone graft in hip surgeries at our department encouraged research and use of bone substitutes, specifically freeze-dried bovine xenografts (Galia et al. 2009a; Macedo et al. 1999). The bovine bone is considered a natural hydroxyapatite with chemical composition, porosity, size and shape similar to the human bone, providing structural and osteoconductive support for new bone formation (Galia et al. 2008). Defatting, cell removal and dehydration by freeze-drying decrease graft antigenicity, maintaining its structural characteristics and protein-mineral matrix. At the end of the process, grafts can be sterilized and easily stored (Kakiuchi et al. 1996). The advantages of xenografts include good availability, easy handling and potentially favorable clinical performance. The xenografts currently used in orthopedic surgery have proven to be safe and reliable (Laurencin and El-Amin 2008).

The main objective of this study was to identify whether there are differences in bony union following subtalar arthrodesis, after calcaneal fracture malunion, with interposition of autologous tricortical iliac crest bone graft or freeze-dried bovine xenograft. Secondary objectives included clinical and radiographic parameters.

## Methods

**Figure 1:**
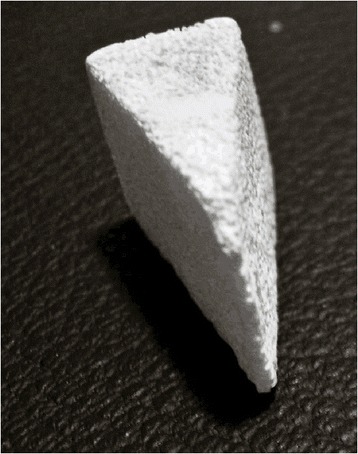
**Freeze-dried bovine xenograft.**

Between September 2006 and October 2007, twelve consecutive non randomized patients underwent subtalar arthrodesis with interposition of a bone graft block for the treatment of calcaneal fracture malunion. Autologous tricortical iliac crest graft (autograft group) was used in six patients (six cases) and freeze-dried cancellous bovine xenograft bone block (xenograft group) in six patients (six cases) (Figure 1). A total of twelve nonrandomized patients were prospectively analyzed with a mean followup of 58.17 weeks (range, 42 to 82 weeks). A bone graft of each type was used in the first two operated cases. Tricortical autograft was then used in the next five cases, and freeze-dried bovine xenograft was subsequently used in the other five cases. The study was approved by the institutional review board (Group for Research and Graduate Studies of Hospital de Clínicas de Porto Alegre) and all patients gave their informed consent.

The sample was composed of patients aged between 20 and 60 years of age with talocalcaneal joint arthrosis after calcaneal fracture who showed significant loss of calcaneal height, limitation of hindfoot range of motion, hindfoot pain, and limitation to perform daily activities. Patients with open calcaneal fracture or osteomyelitis, or those with rheumatic disease, peripheral neuropathy or other diseases or deformities that hinder walking were excluded.

Table 1 summarizes the patients’ data. There was one case of bilateral fracture of the calcaneus in each group, but only one side was operated on in this study. All patients were conservatively treated for the index calcaneal fracture. One case in the autograft group and two cases in the xenograft group had previously undergone lateral calcaneal wall decompression.

**Table 1:** **Description of sample data**

	**Autograft**	**Xenograft**
Sex*	4 F, 2 M	6 M
Age^†^	44.2 years (29–57)	47.2 years (31–56)
Side	4 right	4 right
Smoking	1 patient	3 patients
Time from fracture to surgery^†^	138 weeks (19–330)	134.3 weeks (36–362)
Follow-up^†^**	68 weeks (62–82)	48.3 weeks (38–58)

F: female, M: male. *p = 0.014. **p = 0.001. ^**†**^Mean (minimum-maximum).

Clinical assessment was performed using the American Orthopedic Foot and Ankle Society (AOFAS) Ankle-Hindfoot Scale and the visual analog scale (VAS) for pain pre and postoperatively. Postoperative iliac donor site pain was independently assessed by the visual analog scale.

The following parameters were measured on the lateral weight bearing radiograph of the foot without plaster at final followup: talocalcaneal angle, calcaneal-to-floor angle, talus-first metatarsal angle, talar tilt angle (in degrees), and talar height (in millimeters). Calcaneal width was measured (in millimeters) on the axial heel view of the calcaneus. The opposite side was considered normal for radiographic comparison purposes in all patients but the two with bilateral calcaneal fracture. Clinical and radiographic parameters assessments were done by third year orthopaedic residents with close supervision of the orthopedic surgeon instructor. Two independent blind raters retrospectively examined all lateral radiographs for evidence of solid union of the arthrodesis and graft integration, in addition to signs of loosening of the osteosynthesis material and type of bone graft used (autologous or xenograft). Solid union of the arthrodesis was defined as radiographic union of the talus, bone graft and the calcaneus, and graft integration was defined as the presence of host bone trabeculae replacing graft trabeculae, thus hiding individualization of the graft on radiographs (Easley et al. 2000). Length of time (in weeks) required for solid union of the arthrodesis or graft integration was defined as the first record agreed on by the two raters as long as both raters confirmed radiographic evidence of solid union or graft integration at the final follow-up evaluation. If there was no agreement between raters at the final radiographic evaluation, the case was considered as nonunion or not integrated, regardless of previous assessments. Radiographs were done monthly after surgery until arthrodesis union was considered and at final followup.

The following data were abstracted from the patients’ medical records: duration of surgery, duration of cast immobilization, and postoperative complications.

### Operative technique

Surgery was done by one single foot and ankle surgeon (HC). The patient was positioned supine with a pad under the ipsilateral hip. The lower limb was exsanguinated using an elastic bandage, and a pneumatic tourniquet was placed in the proximal third of the thigh. Calcaneal tendon lengthening was indicated in cases of ankle dorsiflexion less than 10°. An extended lateral L-shaped incision was made to approach the talocalcaneal joint with a single dissection toward the bone plane in all cases. The prominence in the lateral calcaneal wall was then resected. After opening and releasing the talocalcaneal joint capsule, cartilage of the posterior facet of the calcaneus and the talus was removed to expose the subchondral bone. The talocalcaneal space was opened with a distractor until correction of hindfoot bone relationships was achieved, as assessed by intraoperative radiographic control, and a graft block was interposed to fill the space obtained. A single 7.0 mm partially threaded cannulated screw was inserted from the calcaneus to the talus for fixation through the graft block. At the end of the procedure, the tourniquet was released for hemostasis and rinsing with saline, followed by subcutaneous closure with bioabsorbable suture and skin closure with nonabsorbable suture and placement of a plaster splint.

Bone graft was harvested from the anterolateral aspect of the iliac crest. The periosteum was elevated of the internal and external iliac wall, 3 cm posterior to the anterior superior iliac spine. A tricortical bone graft block of the desired size was harvested using an osteotome. Routine closure was performed after rinsing and hemostasis.

The plaster splint was applied for two postoperative weeks until sutures were removed, when the splint was replaced with a non-weight-bearing cast boot. After six weeks, partial weight-bearing was allowed with the cast boot until there was clinical and radiographic evidence of solid union of the arthrodesis, when weight-bearing was allowed as tolerated without immobilization and the motor rehabilitation program started.

Statistical analysis was performed using the following tests: the chi-square test to verify association between variables; the Student *t* test to compare means; Wilcoxon, Mann–Whitney and Levene’s nonparametric tests to compare scores; and the kappa coefficient to assess interrater agreement. The significance level was set at 5%.

## Results

According to the AOFAS Ankle-Hindfoot Scale, there was a significant improvement between preoperative and final followup scores in the autograft and xenograft groups (p = 0.02). There was no statistically significant difference between groups. A statistically significant difference was observed between preoperative and final followup VAS scores in the autograft group (p = 0.028) and in the xenograft group (p = 0.046), with no difference between groups (Table 2).

**Table 2:** **Scores on the American Orthopedic Foot and Ankle Society (AOFAS) Ankle-Hindfoot Scale and visual analog scale (VAS) for pain**

**Group**	**n**	**AOFAS scale** ^**†**^		p*	**VAS for pain** ^**†**^		p*
		**Preop.**	**Postop.**		**Preop.**	**Postop.**	
Autograft	6	37 (11.08)	63.83 (9.58)	0.02	4.73 (2.17)	1.87 (1.33)	0.028
Xenograft	6	38 (14.92)	73.83 (9.54)	0.02	5.53 (1.91)	2.72 (2.4)	0.046
Both	12	37.83 (12.56)	68.33 (10.77)	0.002	5.13 (1.99)	2.29 (2.01)	0.006

*Wilcoxon nonparametric test. ^**†**^Mean (standard deviation).

None of the patients who underwent the procedure to harvest autologous bone graft from the iliac crest had preoperative pain at the donor site. At the final followup, two patients were free of pain and four patients had pain with VAS scores ranging from 0.1 to 0.6. All patients showed a small area of hypoesthesia around the wound, without hindering the performance of daily activities. No major complications were observed in these patients.

Talocalcaneal height showed a statistically significant difference between pre and postoperative values in the autograft group (p = 0.04) and in the xenograft group (p = 0.03). There was a statistically significant difference between pre and postoperative talocalcaneal angle (p = 0.04) and talus-first metatarsal angle (p = 0.04) in the autograft group. In the xenograft group, calcaneal width (p = 0.04) showed a statistically significant difference pre and postoperatively. When each group was evaluated separately, no statistically significant difference was observed between postoperative radiographic parameters in relation to the contralateral side (Table 3).

**Table 3:** **Radiographic parameters**

**Group**	**X-ray**	TC height* ^**†**^	**Calcaneal width** ^**†**^	CF angle* ^**††**^	TC angle* ^**††**^	TFM angle* ^**††**^	**Talar tilt angle** ^**††**^
Autograft	Contralateral	67 (63.5/69.75) [p = 0.18]¶	31.5 (22.5/34.75)	17 (12.25/22.5) [p = 0.11]	41 (32.5/49.5) [p = 0.11]	3 (−6.25/10) [p = 0.18]	21.5 (15.75/28.75) [p = 1.0]
	Preoperative	64 (60/71) [p = 0.04]¶	42 (35/49)¶[p = 0.47]	14 (10.5/21) [p = 0.85]	29 (22/33) [p = 0.04]	12.5 (7.5/17) [p = 0.04]	13.5 (9/18) [p = 0.07]
	Postoperative	69 (63.5/80)	40.5 (36/45.75)	20 (7/23)	36 (30/42)	10 (0/11.5)	18 (16/25)
Xenograft	Contralateral	78 (77.25/79.5) [p = 0.11]	35 (29/35)¶[p = 0.1]	21 (18.5/23.5) [p = 0.14]	46.5 (42.75/51) [p = 0.07]	1.5 (0/3.75) [p = 0.1]	24 (24/28.5) [p = 0.11]
	Preoperative	71.5 (69.5/75) [p = 0.03]	48 (39.75/50.5) [p = 0.04]	13 (11.5/19.75) [p = 0.6]	32 (22/42.5) [p = 0.1]	5.5 (0/13.25) [p = 0.89]	17.5 (9.75/24.75) [p = 0.79]
	Postoperative	76.5 (72.75/80.75)	42 (39/45.25)	15 (10/22)	38 (30.5/44.5)	8.5 (3/10)	18 (16.25/21)
Both	Contralateral	73.5 (66/78) [p = 0.04]	33 (29.5/35) [p = 0.07]	20.5 (14.25/22.75) [p = 0.04]	43.5 (40.5/51) [p = 0.02]	1.5 (−3/8.5) [p = 0.04]	24 (19.5/28.75) [p = 0.11]
	Preoperative	69 (63/72.75) [p = 0.003]	45 (37/50) [p = 0.07]	13 (11.25/19.5) [p = 0.54]	29 (22.5/36) [p = 0.01]	10.5 (0.5/15.25) [p = 0.12]	16 (10/18) [p = 0.06]
	Postoperative	76 (69/79)	42 (37/50)	18 (10/22)	36 (30/44)	10 (4/10)	18 (17/22)

*TC height: talocalcaneal height; CF angle: calcaneal-to-floor angle; TC angle: talocalcaneal angle; TFM angle: talus-first metatarsal angle. ^**†**^Height and width in millimeters. ^**††**^Angle in degrees. ¶ Median (25th percentile/75th percentile) [p value in relation to the postoperative period]: Wilcoxon nonparametric test.

**Figure 2:**
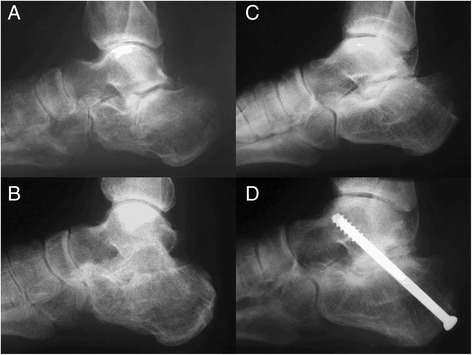
**Lateral radiograph focused on rearfoot showing evidence of solid union of subtalar arthrodesis with use of autologous iliac crest graft (preoperative (A), last followup (B)) and freeze-dried bovine xenograft (preoperative (C), last followup (D)).**

**Figure 3:**
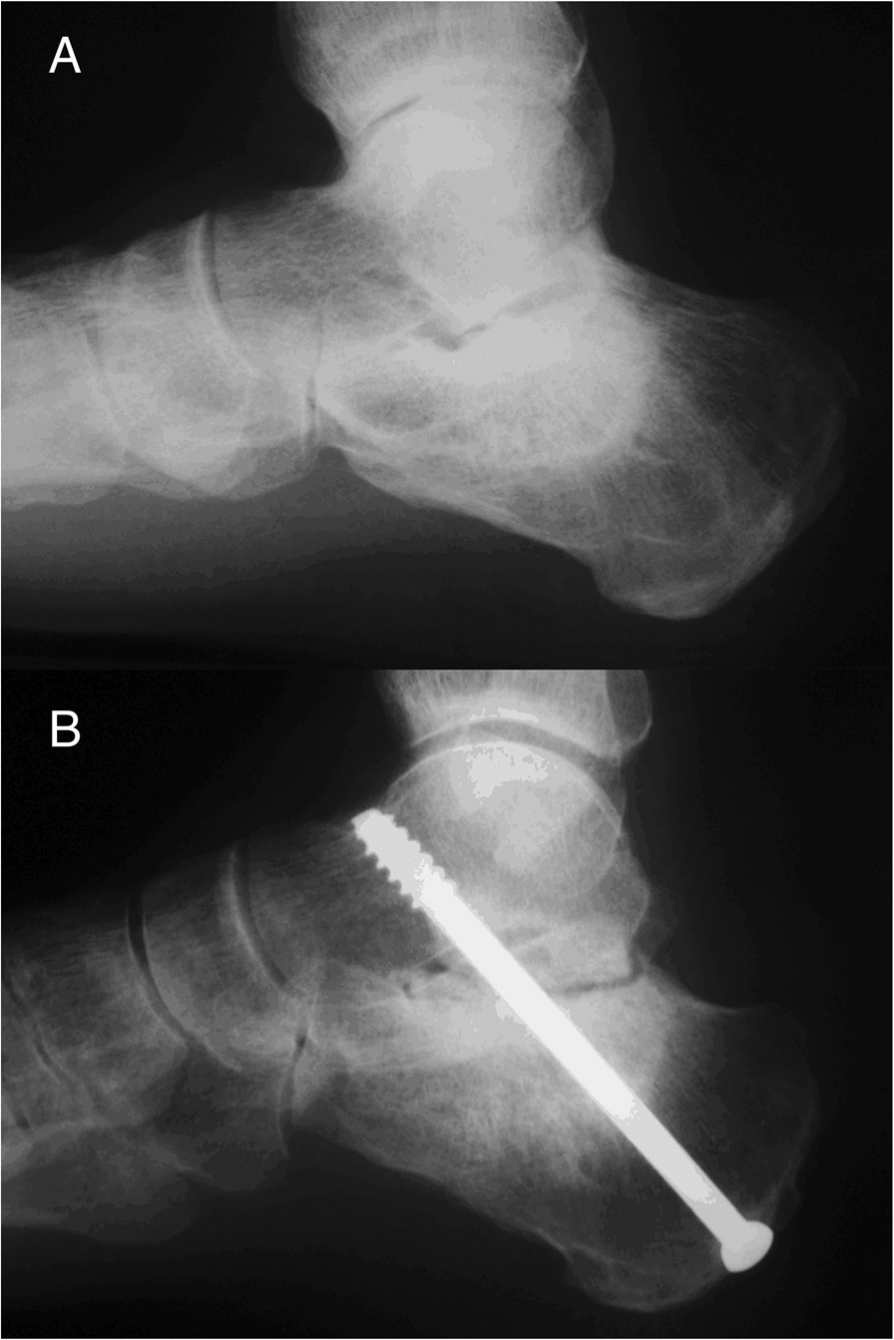
**Preoperative (A) and final postoperative (B) lateral radiograph of subtalar arthrodesis with interposition of freeze-dried bovine xenograft in the case considered as nonunion.**

The retrospective evaluation performed by the two blind raters revealed no statistically significant difference in time to union of the arthrodesis, which was achieved at an average of 5.33 weeks in the autograft group and 8.8 weeks in the xenograft group (p = 0.077). A statistically significant difference was observed for graft integration, which occurred after an average of 10.67 weeks in the autograft group and 28.8 weeks in the xenograft group (p = 0.016) (Table 4). Solid union of the arthrodesis and graft integration were achieved in all six cases in the autograft group and in five out of six cases in the xenograft group (Figure 2). One case in the xenograft group was considered as nonunion (Figure 3). The raters did not identify signs of loosening of the osteosynthesis material in any case. Interrater agreement for identification of the type of graft used was poor (kappa coefficient 0.17) for rater #1 and substantial (kappa coefficient 0.67) for rater #2.

**Table 4:** **Length of time for solid union of the arthrodesis and bone graft integration**

**Group**	Union*	Integration*
Autograft	5.33 (2/8)	10.67 (6/28)
Xenograft	8.8 (6/14)	28.8 (10/38)
p^†^	0.077	0.016

*Mean in weeks (range). ^**†**^P value: Levene’s test.

Mean duration of cast immobilization was 10.33 weeks (range, 8 to 12) in the autograft group and 13.33 weeks (range, 10 to 18) in the xenograft group (p = 0.054). Mean duration of surgery was 114.67 (SD 8.14) minutes in the autograft group and 96.67 (SD 6.83) minutes in the xenograft group (p = 0.002).

At the index procedure the posterior calcaneal tuberosity was removed in one patient of each group because it was still proeminent over the Achilles tendon after realignment of the hindfoot. Calcaneal tendon lengthening was performed in two cases in the autograft group and in three cases in the xenograft group. There was one case of wound dehiscence in the autograft group and two cases in the xenograft group, which were not related with previous surgery. In all patients, wounds were allowed to heal by secondary intention after serial dressing changes, with no need for further surgical intervention. In two patients in the autograft group the screw used for internal fixation was removed after 60 weeks, because its head was prominent in the calcaneus and caused discomfort wearing regular shoes. One patient in the autograft group persisted with a varus hindfoot and underwent a calcaneus valgus osteotomy with cannulated screw fixation to restore hindfoot alignment. One patient in the autograft group developed complex regional pain syndrome (CRPS) in the immediate postoperative period and had the cannulated screw removed in the 18th week, since it appeared to be prominent dorsally into the body of the talus. The patient continued with pain and daily activities restriction afterwards, despite use of pain medication, shoe modification and physical therapy. In one patient in the xenograft group a bony prominence in the medial wall of the calcaneus was resected in the 54th week, as it was causing friction against the flexor hallucis longus tendon, which alleviated symptoms promptly. There was involvement of the sural nerve with complaints of paresthesia and hypoesthesia on the lateral aspect of the hindfoot in four patients in the autograft group and in three in the xenograft group. Five out of six patients in the autograft group and four out of six patients in the xenograft group complained of diffuse pain in the hindfoot on physical examination at the final followup. None of the patients that entered the study were excluded from data analisys.

Based on the data obtained in our study, a power calculation indicated that eleven cases in each group would be required to assess time to union of the arthrodesis and five cases in each group to assess time to graft integration with a study power of 80% at a significance level of 5%.

## Discussion

In the surgical treatment of sequelae of calcaneal fractures, all possible causes of pain should be considered and the anatomy of the hindfoot restored (Nickisch and Anderson 2006; Paley and Hall 1993; Savva and Saxby 2007). Carr *et al.* (Carr et al. 1988) described the subtalar arthrodesis with interposition of bone graft to restore normal alignment of the hindfoot and thus achieve functional improvement, since they believed that loss of talocalcaneal height leads to painful anterior tibiotalar impingement. Myerson and Quill (Myerson and Quill 1993) indicate this type of arthrodesis when there is more than 8 mm loss of talocalcaneal height in relation to the contralateral side or when there is evidence of anterior ankle impingement demonstrated by a talar tilt angle less than 20°. Chandler *et al*. (Chandler et al. 1999) suggest that this type of arthrodesis should be performed in cases of anterior ankle pain and ankle dorsiflexion less than 10°.

Radiographic evaluation of union of the arthrodesis and graft integration is subjective. The length of time required for union of the arthrodesis was higher in the xenograft group (average 8.8 weeks) then in the autograft group (average 5.33), although not statistically significant (p = 0.077), as evaluated by two independent blind raters. Our patients were placed in a non-weight-bearing cast for 6 weeks followed by partial weight-bearing until there was clinical and radiographic evidence of union. Mean immobilization time was 10.33 weeks in the autograft group and 13.33 weeks in the xenograft group, which difference was not statistically significant. The mean immobilization time employed by the authors was longer than the mean time required for union of the arthrodesis according to the radiographic criteria used by both blind raters. Time to arthrodesis union considered as the time of immobilization favors the results described in the literature that range from 12 to 20 weeks (mean, 14 weeks) with fresh-frozen allografts and from 10 to 30 weeks with autografts for a solid union to be obtained (Bednarz et al. 1997; Easley et al. 2000; Trnka et al. 2001).

Other factors that may be related to the occurrence of nonunion include smoking and presence of avascular necrosis of bone (Clare et al. 2005; Easley et al. 2000; Trnka et al. 2001). In the present study, the only case of nonunion occurred in one of the three smokers in the xenograft group that also showed signs of calcaneal bone sclerosis, suggestive of bone necrosis, at final x-ray evaluation (Figure 3).

Time to graft integration, 28.8 weeks in the xenograft group and 10.67 weeks in the autograft group, had a statistically significant difference (p = 0.016). Freeze-dried bone grafts, due to the process which they undergo, are considered to have osteoconductive properties, with no osteoinductive or osteogenic characteristics, which might delay graft integration (Hartl et al. 2004). Even fresh-frozen allograft need 24 to 48 weeks to revascularizate and integrate (Garras et al. 2008; Myerson et al. 2005). The association of osteoinductive properties to freeze-dried xenograft bones, to maybe increase union rate and accelerate graft integration, is open to further research.

The AOFAS scale is a validated, widely accepted rating scale (Schepers et al. 2008). In our series, patients showed a statistically significant improvement as assessed by the AOFAS scale or VAS for pain. AOFAS scores improved 30.5 points, from a preoperative average of 37.8 to 68.3 points postoperatively (p = 0.002), and VAS scores improved 2.84, from a preoperative average of 5.13 to 2.29 postoperatively (p = 0.006). These values are close to those described in the literature, with the improvement in AOFAS scores ranging from 32 to 50 points, from a preoperative average between 20 and 40 points to an average between 69 and 75 points postoperatively (Amendola and Lammens 1996; Bednarz et al. 1997; Garras et al. 2008; Rammelt et al. 2004; Trnka et al. 2001). VAS scores range from a preoperative average between 6.07 and 8.1 to an average between 2.0 and 2.56 postoperatively (Amendola and Lammens 1996; Trnka et al. 2001). Nevertheless, all six patients in the autograft group and four patients in the xenograft group complained of pain in the operated foot at the final followup. Persistent residual pain after arthrodesis with interpositional graft was described by Clare *et al*. (Clare et al. 2005) in 64% of 45 patients after a mean followup of 5.3 years and by Marti *et al.* (Marti et al. 1999) in 82% of 19 patients after a mean followup of nine years. We agree with Chan and Alexander (Chan and Alexander 1997) who stated that, although pain relief is neither complete nor universal, patient satisfaction is high.

The analysis of radiographic parameters revealed a statistically significant improvement in talocalcaneal height from pre to postoperative values in both groups. This improvement, however, was not sufficient to equal the talocalcaneal height obtained postoperatively with the contralateral side, regarded as the normal parameter for the patient, with a persisting statistically significant difference (p = 0.04) when data from both groups were analyzed together. However, when groups were evaluated separately, no significant difference was found between postoperative and contralateral side values. This may be due to a small number of cases in each group. The talar tilt angle did not show significant differences at any time point. The calcaneus was found to be significantly narrower, as measured by its width, only between the pre and postoperative periods in the xenograft group (Table 3). The amount of correction of these radiographic variables is not uniform in the literature (Burton et al. 1998; Pollard and Schuberth 2008; Rammelt et al. 2004) and has not shown, in some studies, correlation with clinical findings (Chandler et al. 1999; Flemister et al. 2000). Chen *et al.* (Chen et al. 1998) observed that correction of the talocalcaneal height was achieved in 80.1% of cases with good functional results and in only 47.6% of cases with fair results. Marti *et al.* (Marti et al. 1999) did not find correlation between radiological measurements and clinical findings, except for the height of the calcaneal fat pad. The time from fracture to arthrodesis is also indicated by some authors as a factor that may influence the amount of correction possible due to retraction of periarticular tissues of the hindfoot (Amendola and Lammens 1996; Burton et al. 1998; Rammelt et al. 2004). In our series, the mean time from fracture to arthrodesis was 136.17 weeks, which may have influenced the non-correction of some radiographic parameters.

Solid union of subtalar arthrodesis with interposition of autologous iliac crest bone graft in the treatment of malunited fractures of the calcaneus is described in the literature as occurring in 86 to 100% of cases (Amendola and Lammens 1996; Bednarz et al. 1997; Burton et al. 1998; Chan and Alexander 1997; Chen et al. 1998; Myerson and Quill 1993; Rammelt et al. 2004; Thermann et al. 1999). The procedure to harvest bone graft from the iliac crest can cause complications in up to 49% of cases (Finkemeier 2002; Seiler and Johnson 2000). This complication rate is lower for foot arthrodesis cases, affecting only up to 6% (Burton et al. 1998; Trnka et al. 2001). Because it is a surgical procedure that adds the possibility of comorbidities, iliac crest bone graft harvesting is not always easily accepted by patients (Pollard and Schuberth 2008), and is also likely to result in longer operative time and higher costs (Scranton 2002). The duration of surgery was 18% higher in the group undergoing iliac crest bone graft harvesting which was statistically significant (p = 0.002).

Other sources of bone graft have been described in the literature (Kalamchi and Evans 1977). Clare *et al.* (Clare et al. 2005) described good results in 40 patients (45 feet) who underwent subtalar arthrodesis with interposition of autologous bone graft harvested from the lateral wall of the calcaneus, achieving a 93.5% union rate. The use of allografts in this situation showed, in some studies, solid union in only 20 to 40% of cases (Easley et al. 2000; Trnka et al. 2001). Other authors, however, have reported better results using allografts, with union rates similar to those achieved using autografts (Myerson et al. 2005; Pollard and Schuberth 2008; Scranton 2002). Nickisch and Anderson (Nickisch and Anderson 2006) indicate the use of allografts when bone grafts over 1 cm in height are needed for interposition in the subtalar arthrodesis. Garras *et al.* (Garras et al. 2008) described a series of 21 cases using fresh-frozen allografts for interposition in the subtalar arthrodesis with 90.5% union rate, although, in seven cases, grafts were used in association with platelet-rich plasma.

Due to limited availability of fresh-frozen allografts in our department, we have started research and development of a bone bank with freeze-dried bovine bone grafts (Galia et al. 2009a; Galia et al. 2009b; Macedo et al. 1999). In the medical literature, there are only a few studies describing the use of xenografts (Laurencin and El-Amin 2008; Zabeu and Mercadante 2008). Their use in different situations has yielded good results concerning incorporation of the graft and union of the arthrodesis (Malca et al. 1996; Salama 1983; Säveland et al. 1994). In the only study found in the literature on the use of bone xenograft in arthrodeses of the foot and ankle, Thompson *et al.* (Thompson et al. 2002) described five cases that developed nonunion and required further surgical intervention using autografts to achieve solid union of the arthrodesis. Those authors contraindicated the use of xenografts. In our experience, solid union of subtalar arthrodesis with interposition of freeze-dried xenograft bone block was achieved in five out of six cases (83% union rate) and in all six cases (100% union rate) with autologous tricortical iliac crest bone graft after a mean followup of 58.17 weeks.

The use of an extended lateral L-shaped approach allows adequate visualization of the peroneal tendons and calcaneocuboid and subtalar joints, also allowing the lateral calcaneal wall prominence to be easily resected (Clare et al. 2005). However, this approach poses a potential risk for incision closure, mainly when grafts of great height are used, therefore more vertical incisions are preferred in these cases (Pollard and Schuberth 2008). In our study, three cases (25%) developed wound dehiscence. Clare *et al.* (Clare et al. 2005), using this approach, reported the occurrence of wound healing problems in 24% of 45 operated feet. Another common complication is sural nerve injury, which may occur in up to 17% of cases (Rammelt et al. 2004), and may result from direct injury or stretch injury due to increased talocalcaneal height. (Garras et al. 2008) Complaints related to sural nerve injury in our series accounted for seven cases (58%), a complaint rate higher than expected, which might be related to the surgical learning curve for this technique.

## Conclusion

Solid union of subtalar arthrodesis after calcaneal fracture was achieved in all six cases that we used autologous iliac crest graft and in five out of six cases that we used freeze-dried bovine xenograft. There was no statistically significant difference in the length of time required for solid union to occur with the numbers available. Time to integration of freeze-dried bovine xenografts was statistically higher than that of autologous iliac crest grafts. The limited number of patients is a weakness of this study and the results are preliminary, but promising, which stimulates further research using freeze-dried bovine xenografts as a bone substitute.
